# Association of Human Milk Fortifier and Feeding Intolerance in Preterm Infants: A Cohort Study about Fortification Strategies in Southwest China

**DOI:** 10.3390/nu14214610

**Published:** 2022-11-02

**Authors:** Ting Zhang, Huan Luo, Hua Wang, Dezhi Mu

**Affiliations:** 1 Department of Pediatrics, West China Second University Hospital, Sichuan University, Chengdu 610041, China; 2 Key Laboratory of Birth Defects and Related Diseases of Women and Children, Ministry of Education, Sichuan University, Chengdu 610041, China

**Keywords:** breast milk, southwest China, enteric nutrition, feeding intolerance, human milk fortifier, preterm infant, smooth fitting curve, threshold effect

## Abstract

Background: The present strategy of administering human milk fortifier (HMF) in southwest China (swC) is mainly based on European and American populations’ guidelines. Additionally, some southwest Chinese preterm infants have been observed to develop feeding intolerance (FI) after administration of HMF. In order to develop adapted southwest Chinese guidelines for the administration of HMF to preterm infants and improve fortification strategies, a retrospective cohort study was performed to explore the association of the use of HMF and FI. Objective: To explore the association between HMF and FI in preterm infants and provide recommendations for its use in swC. Methods: This cohort study included 298 preterm infants from West China Second University Hospital. Maternal and infant clinical data were collected from electronic patient records. The infant cohort was divided into two groups based on the use/nonuse of HMF. The association between HMF and FI was evaluated using multivariate analysis. Nonlinear relationships and threshold effects were evaluated using generalized additive models and two-piecewise linear regression models. Results: The multivariate analysis confirmed that there is no significant association between HMF use and FI, but significant risk factors for FI include early HMF initiation (*p* = 0.02), full-strength HMF initiation (*p* = 0.04), and fast HMF supplementation rates (*p* = 0.004). Through smooth curve fitting and threshold effect analysis, we found that two inflection points, an initial concentration of HMF > 24 mg/mL and a HMF supplementation rate > 12.5 mg/mL/d, significantly increased FI risk. Conclusions: Routine HMF fortification can be safely used in preterm infants with gestational age < 32 wk or birth weight < 1500 g in swC, and we advise initiating fortification when enteral milk intake reaches 100 mL/kg/day, with an HMF concentration of 1:50 and if tolerated, increase to 1:25 more than 38 h. The recommended HMF supplementation rate differs from current guidelines and provides evidence for developing southwest Chinese guidelines. A prospective trial is needed in order to validate this proposal.

## 1. Introduction

The survival rate of preterm infants has increased significantly in recent years, due to the development of various medical treatments and life support technologies. However, promptly and safely achieving total enteral nutrition in preterm infants is still a significant challenge for neonatologists. Human breast milk is recommended as the optimal food choice for infants for its nutritional and immunological advantages. However, breast milk alone is insufficient for preterm infants who need additional nutrients to meet their growth demands [1]. Furthermore, the nutrients in breast milk are not stable and can vary between individuals, over time, and with maternal diet [2,3,4]. When human breast milk cannot meet the needs of preterm infants, human milk fortifier (HMF) is used as an additional nutritional supplement [5,6,7].

However, the usage of HMF still needs to be clear-cut. Current guidelines and consensus have different ideas regarding who is eligible for HMF [8,9,10,11,12]. As for when and how to use HMF, the Consensus Statement [8] recommends that HMF should be started as standard fortification if infants do not grow appropriately. The American Academy of Pediatrics (AAP) indicates that the optimal timing of fortification remains unclear [11]. Only the Canadian Guideline provides complete instructions: start fortification when enteral intake reaches 100 mL/kg/day, start at a concentration of 1:50, and if this is tolerated for 48 h, increase to 1:25 [9]. Based on the differences between the recommendations and finding that some preterm infants at our clinic would develop feeding intolerance (FI) symptoms after being administered HMF, we decided to conduct a study to explore two questions: Is the current HMF fortification strategy appropriate for preterm infants in **Copyright:** © 2022southwest China? Is there a relationship between the use of HMF and FI in preterm infants?

Our present study differs from most previous studies on HMF, which either focus on the effects of HMF on preterm infant growth and development rate or compare different HMF varieties. The objective of our study was not only to evaluate the relationship between the use of HMF and FI development, but also to provide recommendations for its use in southwest China.

## 2. Methods

### 2.1. Study Design

This retrospective cohort study was conducted at the West China Second University Hospital, Sichuan University. The infant cohort was divided into two groups based on the use/nonuse of HMF. The primary outcome measure was FI diagnosis.

### 2.2. Participants

Data were retrospectively collected from the inpatient electronic medical records of infants admitted to the neonatal intensive care unit (NICU) between June 2015 and November 2018. The inclusion criteria for this study were as follows: birth weight (BW) < 1500 g or gestational age (GA) < 32 wk, admitted to the NICU within 24 h after birth, and received maternal breast milk for enteral feeding during NICU hospitalization. The exclusion criteria for this study were as follows: serious congenital malformation or metabolic disease, gastrointestinal disease other than FI that prevented achieving total enteral nutrition, and hospitalization in the NICU < 14 days.

**Figure 1:**
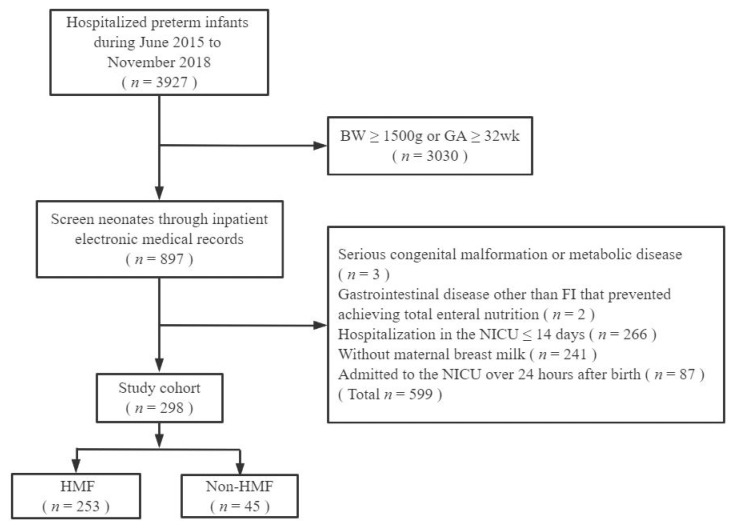
Patient flow diagram. BW, birth weight; FI, feeding intolerance; GA, gestational age; HMF, human milk fortifier; NICU, neonatal intensive care unit.

Infants who met all study criteria were divided into two groups: infants who received HMF supplementation (HMF), and infants who did not receive HMF supplementation (non-HMF). The patient flow chart is shown in Figure 1. All included data were anonymous; therefore, informed consent was not required.

### 2.3. F1 Diagnosis

Although FI has been widely studied, there is still no uniform definition or diagnostic test for this condition. Considering the diagnostic guidelines mentioned by other researchers [13,14,15], this study used the following criteria: feeding volume maintained or decreased >3 days; gastric residual volume greater than half of the previous feeding; and bloating or vomiting. FI was diagnosed if any of the above criteria were met.

### 2.4. Equations and Definitions

The following equations and definitions were used to assess infant feeding:

Milk volume = amount of milk a day/weight of that day (mL/kg/d)

Rate of milk supplementation = (terminal milk volume − initial milk volume)/days (ml/kg/d)

The terminal milk volume was defined as the volume of milk fed when the neonate achieved total enteral nutrition if FI did not occur during the feeding process. Total enteral nutrition was defined as enteral feeding of 150–180 mL/kg/d [9]. If FI did occur during the feeding process, the terminal milk volume was defined as the milk volume fed when FI occurred.

Human milk (HM) volume when HMF supplement was defined as HM volume when the infant received HMF for the first time. If an infant never received HMF during hospitalization, the value of HM volume with HMF supplement was coded as NA. Early fortification was defined as the HM volume when HMF supplement < 100 mL/kg/d, and late fortification was defined as the HM volume when HMF supplement ≥ 100 mL/kg/d [16].

The initial concentration of HMF was calculated by the amount of HMF divided by the volume of HM when HMF was given the first time, and the measurement unit was mg/mL.

Rate of HMF supplementation = (terminal HMF concentration − initial HMF concentration)/days (mg/mL/d)

The terminal HMF concentration was defined as the concentration fed when HMF was administered for the final time, if FI did not occur during the process. If FI occurred during the process, the terminal HMF concentration was defined as the concentration administered when FI occurred. The change in rate of HMF supplementation was negative in case of increasing amounts of milk without increasing HMF supplementation. If an infant never received HMF during hospitalization, the value of the initial concentration of HMF and rate of HMF supplementation was 0.

Early blood transfusions were defined as blood transfusions performed within two weeks after birth, whereas late blood transfusions were defined as blood transfusions performed more than two weeks after birth.

### 2.5. HMF

The HMF used in this study was FM85 (Nestléstrasse 1, 3510 Konolfingen, Switzerland, powdered, bovine milk-derived, and moderately hydrolyzed). And the breast milk used for fortification came from the infants’ own mothers. A total of 4 g HMF was added for every 100 mL of breast milk; as a result, the concentration of full-strength fortified milk was 1:25 or 40 mg/mL, and the concentration of half-strength fortified milk was 1:50 or 20 mg/mL. The osmotic pressure of full-strength fortified milk was 339 mOsm/L. The nutrition information of HMF in this study is provided in Table 1.

Since there are no guidelines for the use of HMF in China, the decision about whether to start or when to start HMF and how to administer it were made by pediatricians depending on relevant recommendations, their clinical experience, and the individual situation of preterm infants. In this retrospective cohort study, we analyzed the associations between the use of HMF and FI in order to develop appropriate guidelines.

### 2.6. Statistical Analysis

All analyses were performed using R software version 3.4.3 (The R Foundation for Statistical Computing, Vienna, Austria) and EmpowerStats (X&Y Solutions, Inc., Boston, MA, USA). Continuous variables with normal distribution were reported as mean ± SD, continuous variables with non-normal distribution were reported as median and range, and categorical variables were reported as count and percentage. Descriptive analyses to evaluate differences between the two groups (HMF vs. non-HMF) included *t*-tests for continuous variables with normal distribution, nonparametric statistic tests for continuous variables with not normal distribution, and *X*^2^ tests for categorical variables.

The relationships between the use of HMF (nonuse vs. use, early vs. late fortification, initial concentration of HMF, and rate of HMF supplementation) and the appearance of FI were explored using univariate logistic regression analyses. This process was followed by a multivariate logistic regression analysis controlling for covariates, which were selected based on their association with the outcome of interest or a change in effect estimate > 10%. A forest map was generated based on the results of multivariate analyses to visually display the relationship between the use of HMF and FI. The generalized additive models were used to investigate nonlinear relationships between the initial concentration of HMF and FI, and rate of HMF supplementation and FI. Two-piecewise linear regression models were also used to examine the threshold effects according to the smoothing plots. In all analyses, *p* < 0.05 was considered statistically significant. Statistical outliers were excluded from the analysis, dummy variables and mean value imputation were used to indicate missing covariate values.

## 3. Results

A total of 298 preterm infants were included in this study. There were 253 (84.9%) infants in the HMF group and 45 (15.1%) infants in the non-HMF group. FI occurred in a total of 160 cases. Significant differences were observed in patient BW, GA, 1-min Apgar score, admission temperature, rate of milk supplementation, respiratory support, pulmonary surfactant (PS), caffeine, and patent ductus arteriosus (PDA) between the two groups (Table 2). Among infants in the HMF group, 14 (5.53%) infants received early fortification, while 239 (94.47%) received late fortification. Half-strength fortified milk was used for 265 (89.2%) infants while full-strength fortified milk was used for 32 (10.8%) infants. The range of HMF supplementation rates was −2 to 27 mg/mL/d and was divided into three categories (−2 to 2 mg/mL/d, 2 to 5 mg/mL/d, 5 to 27 mg/mL/d) to facilitate logistic regression analysis.

**Figure 2:**
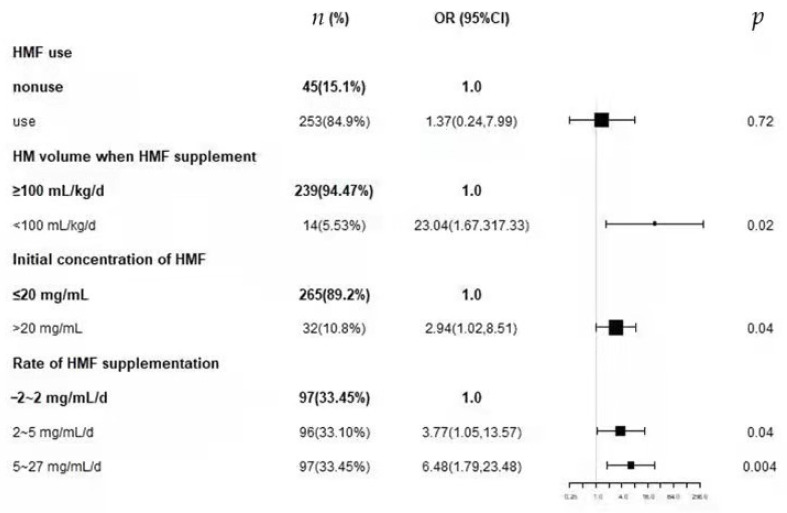
Forest plots for multivariate logistic regressions of the use of HMF on FI. FI, feeding intolerance; HM, human milk; HMF, human milk fortifier. Results on the right side reflect an increase in FI.

Table 3, Table 4, Table 5 and Table 6 provide the results of univariate and multivariate logistic regressions that assessed the association between different factors of the use of HMF and FI. In Table 3, we can see no significant association between HMF use and FI in either the unadjusted model or adjusted models. Table 4 shows that early fortification significantly affects FI in both the unadjusted and adjusted models. In the unadjusted model, the odds ratio (OR) is 5.57 and *p* = 0.03, while the OR is 23.04 and *p* = 0.02 in the adjusted model. Table 5 reveals that although there is no significant association between the initial concentration of HMF and FI in the unadjusted model (OR 1.49; *p* = 0.30), the two factors have a significant association after being adjusted (OR 2.94; *p* = 0.04). Table 6 shows a similar relationship between the rate of HMF supplementation and FI. In the unadjusted model, there is no significant association between the two factors (OR 1.13; *p* = 0.68 and OR 0.90; *p* = 0.71), but after being adjusted, the association became significant (OR 3.77; *p* = 0.04 and OR 6.48; *p* = 0.004). The results of these multivariate analyses were visually displayed on a forest map (Figure 2).

**Figure 3:**
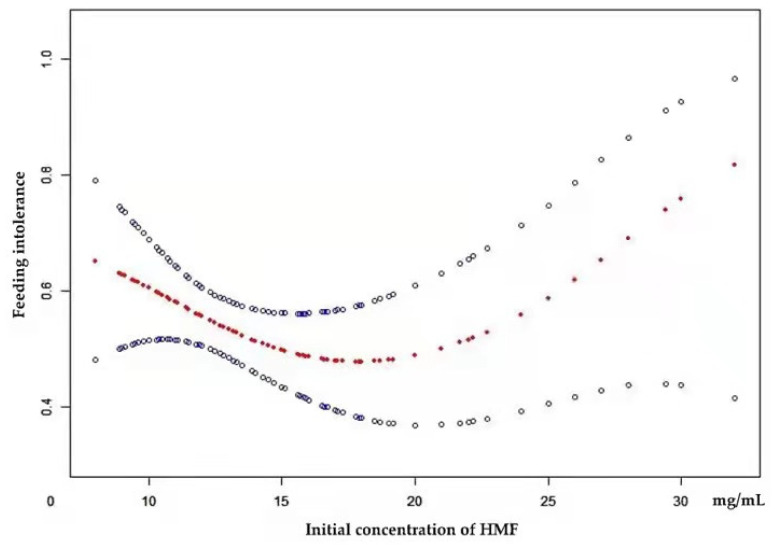
Estimated probability of experiencing feeding intolerance (FI) by initial concentration of human milk fortifier (HMF), based on a generalized additive model controlling for birth weight (BW), rate of milk supplementation, and HM volume when HMF supplement. The red curve in the middle is the spline smoothing, and the blue curve on both sides represents the 95% confidence interval.

Figure 3 shows a nonlinear relationship between the initial concentration of HMF and FI. The smooth curve fitting was performed after adjusting for BW, rate of milk supplementation, and HM volume when HMF supplementation. The resultant curve exhibited a two-stage change and an inflection point (K = 24) (Table 7). On the left of the inflection point, the OR (95% confidence interval [CI]) and *p*-value were 0.93 (0.86–1.01) and 0.10, respectively. On the right of the inflection point, the OR (95% CI) and *p*-value were 1.68 (1.01–2.78) and 0.04, respectively. The difference was statistically significant compared to the linear model (*p* = 0.01; log-likelihood ratio test).

**Figure 4:**
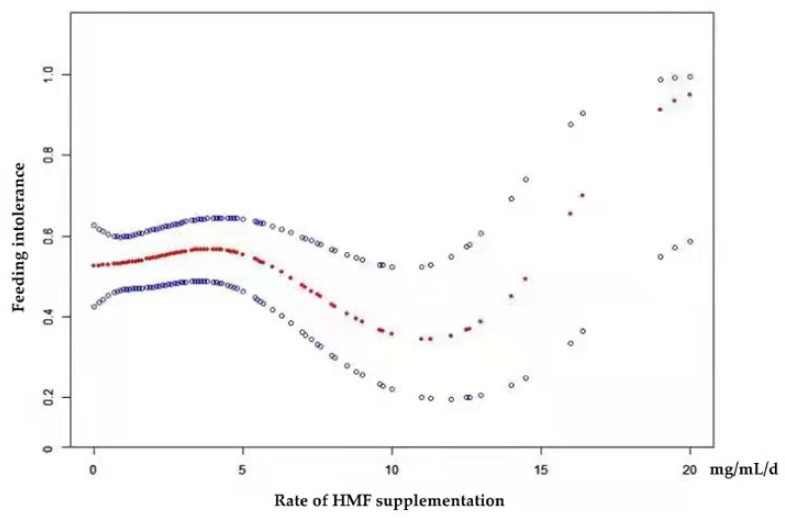
Estimated probability of experiencing feeding intolerance (FI) by rate of human milk fortifier (HMF) supplementation, based on a generalized additive model controlling for gestational age (GA); birth weight (BW); admission temperature; initial concentration of HMF. The red curve in the middle is the spline smoothing, and the blue curve on both sides represents the 95% confidence interval.

As shown in Figure 4, the rate of HMF supplementation also had a non-linear relationship with FI after adjusting for BW, GA, admission temperature, and initial concentration of HMF. By using a two-piecewise linear regression model, the inflection point was revealed to be 12.5 (Table 8). On the left of the inflection point, the OR (95% CI) and *p*-value were 0.93 (0.86, 1.01) and 0.07, respectively. On the right of the inflection point, the OR (95% CI) and *p*-value were 1.74 (1.08, 2.81) and 0.02, respectively. The difference was statistically significant compared with the linear model. (*p* = 0.002; log-likelihood ratio test).

## 4. Discussion

Although HMF is increasing in popularity, there are gaps in the use of HMF between developed and developing countries. In the 1970s, developed countries began adding microelements (such as iron) in breast milk for preterm infants [17,18,19,20], and in the 1980s, studies were being conducted on the use of HMF [21,22,23,24]. On the other hand, HMF was introduced in China in the 2000s, but until the 2010s, there was limited evidence for using HMF on preterm infants [25,26,27,28]. Although some guidelines guide the use of HMF clinical practice, their recommendations are not consistent. In addition, there is only a consensus to use HMF in China but no available guidelines to aid its use. Therefore, research on how to use HMF based on statistical analyses of clinical data is needed in China.

HMF supplementation is expected to promote growth in premature infants. However, as with many other interventions, it has adverse effects [29,30]. For example, HMF can increase the osmolarity of breast milk, and the increase in osmolarity combined with the protein component of HMF can result in delayed gastric emptying [31,32]. Despite the known gastrointestinal side effects of HMF, no previous studies have demonstrated an association between HMF and FI [29]. The univariate and multivariate logistic regression analyses found no association between HMF use and FI in this study. However, significant associations were found between early initiation of HMF, initial concentration of HMF, HMF supplementation rate, and FI.

There is debate on when to administer HMF to premature infants. An earlier systematic review stated that there was no difference between early and late fortification [16], while another study showed that starting HMF later was better than starting it earlier [33]. The Canadian Guidelines recommended that fortification should start once enteral intake reaches 100 mL/kg/d [9]. Alternatively, the Chinese Consensus [34] recommended starting HMF when an infant’s breastfeeding intake reaches 50–80 mL/kg/d. The results of the present study revealed that early HMF fortification significantly increased the risk of FI in preterm infants when compared to late fortification (OR 23.04; *p* = 0.02); therefore, this study supports starting HMF supplementation when enteral intake is equal or greater than 100 mL/kg/d to prevent FI.

Regarding the initial concentration of HMF and the rate of HMF supplementation, the Canadian Guidelines recommended starting HMF supplementation at a concentration of 1:50 for 48 h, then increasing the concentration to 1:25 if the preterm infant tolerated it [9]. The Chinese Consensus recommended starting HMF supplementation with half-strength fortification and increasing to full-strength fortification within 3~5 d if infant tolerance allowed it [34].

Multivariate logistic regression analyses of the present study illustrated that full-strength fortification could increase the risk of FI compared with half-strength fortification. The results of curve fitting (Figure 3) and two-piecewise linear regression also revealed that the risk of FI increased when the initial concentration of HMF was greater than 24 mg/mL, a concentration that is similar to half-strength fortified milk (20 mg/mL). This finding accurately supports the conclusion that HMF administration should be started at half-strength HMF to prevent FI.

However, the recommended rate of HMF supplementation in the present study is inconsistent with the Canadian Guidelines or the Chinese Consensus. The results of multivariate logistic regression illustrated that the rate of HMF supplementation was an independent risk factor for FI. Curve fitting and two-piecewise linear regression results also found a nonlinear relationship and threshold effect between the two factors. When the rate of HMF supplementation was greater than 12.5 mg/mL/d, the risk of FI increased significantly. In other words, if the time from half-strength fortification to full-strength fortification was less than 38 h, the risk of FI in preterm infants significantly increased. The recommended rate of HMF supplementation is faster than the rate recommended by the Canadian Guidelines and the Chinese Consensus.

The present study differs from previous studies on HMF because it explored the nonlinear relationship and threshold effect between the initial concentration of HMF and FI and the rate of HMF supplementation and FI. These results can help us develop a strategy for the precise administration of HMF in preterm infants in southwest China. Given the retrospective nature of this study, observation bias may have been reduced, but missing data cannot be avoided. There were a lot of missing values about body measurements, especially head circumference and height measurements. A prospective study is needed in the future to collect anthropometric data in detail to explore the association of the use of HMF with the development of preterm infants. There are also other limitations in this study. Since it was a retrospective observational design without randomization, there were differences between the two groups, although we adjusted for baseline characteristics of the patients, unmeasured confounders could have remained.

Moreover, the present study was conducted in a single center in southwest China and based on bovine milk-derived HMF. The results may be just suitable for southwest Chinese preterm infants and can be used to guide the use of bovine milk-derived HMF. The results of this study will need to be validated prospectively, taking into account hard endpoints such as weight evolution, duration of hospital stay, and more. More extensive, multicenter, and prospective research with infants of different races is needed in the future.

In summary, the safety of HMF was reaffirmed in the present study. However, early HMF initiation, full-strength HMF initiation, and high rates of HMF supplementation were associated with FI development. Based on the results of our study in southwest China, we recommended the routine use of HMF in premature infants with GA < 32 wk or BW < 1500 g, which should be initiated when the consumed HM volume is equal to or greater than 100 mL/kg/d at a concentration of 1:50 (half-strength fortification). If this feeding plan is tolerated, HMF concentration can be increased to 1:25 (full-strength fortification) for more than 38 h. This recommendation differs from the Canadian Guidelines of 48 h or the 3–5 d recommendation by the Chinese Consensus.

## Figures and Tables

**Table 1:** Nutrition information of human milk fortifier in this study.

Nutrient Content	Unit	Moderately Hydrolyzed	
		/100 mL	/100 kcal
Energy	kcal/100 mL	85.44	100.00
Protein	g	3.04	3.56
Lipid	g	4.22	4.96
Linoleic acid	mg	516	604
α-linolenic acid	mg	45.6	53.4
Linoleic acid: α- linolenic acid	-	11	11
ARA	mg	20.00	23.41
DHA	mg	15.8	18.5
Carbohydrate	g	8.596	10.061
Vitamin A	ugRE	347.2	406.4
Vitamin D	ug	3.72	4.35
Vitamin E	mg α-ET	3.98	4.66
Vitamin K	ug	9.4	11
Vitamin B1	ug	148.9	174.3
Vitamin B2	ug	211	247
Vitamin B6	ug	126	148
Vitamin B12	ug	0.2	0.24
Nicotinic acid	ug	1610	1884
Folic acid	ug	40.5	47.4
Acid regurgitation	ug	870	1018
Biotin	ug	3.78	4.42
Vitamin C	mg	23	26.92
Choline	mg	8	9.36
Inositol	mg	4	4.68
Taurine	mg	5.8	6.79
Carnitine	mg	1.98	2.32
L- carnitine	mg	-	-
Ca	mg	100.6	117.7
P	mg	58.3	68.2
Ca/P	-	1.73	1.73
Mg	mg	7.3	8.5
Fe	mg	1.89	2.21
Zn	mg	1.31	1.53
Mn	ug	6.67	7.91
Cu	ug	90	105.3
I	ug	30.6	35.82
Se	ug	4.8	5.62
Na	mg	64.72	75.75
K	mg	98	115
Cl	mg	90.1	105.5
Osmotic pressure	mOsm/L	339	
Degree of hydrolysis	-	moderately hydrolyzed	
Suggested blanking method	-	4 g + 100 mL human milk	

**Table 2:** Maternal pregnancy and clinical characteristic of infants, by HMF or non-HMF. Dates (2015–2018).

	HMF	Non-HMF	*p* Value
Variables	(*n* = 253)	(*n* = 45)	
Placenta previa	16 (6.32%)	6 (13.33%)	0.10
Placental abruption	13 (5.14%)	0 (0.00%)	0.12
Glucocorticoid	172 (67.98%)	32 (71.11%)	0.71
Gestational diabetes mellitus	54 (21.34%)	10 (22.22%)	0.90
Gestational age (wk)	29.91 ± 1.95	30.70 ± 1.33	0.01
Birth weight (g)	1272.17 ± 243.77	1410.22 ± 252.36	<0.001
1-min Apgar score	7.76 ± 2.13	8.47 ± 2.03	0.04
5-min Apgar score	9.11 ± 1.33	9.38 ± 0.86	0.19
Admission temperature	36.16 ± 0.58	36.39 ± 0.53	0.01
Sex			0.45
Female	105 (41.50%)	16 (35.56%)	
Male	148 (58.50%)	29 (64.44%)	
Blood transfusion			0.68
No transfusion	134 (53.39%)	21 (46.67%)	
Early transfusion	55 (21.91%)	12 (26.67%)	
Late transfusion	62 (24.70%)	12 (26.67%)	
Number of blood transfusion			0.88
≤3	243 (96.05%)	43 (95.56%)	
>3	10 (3.95%)	2 (4.44%)	
Age to start oral feeding (d)	1.00 (1.00–14.00)	1.00 (1.00–7.00)	0.55
Rate of milk supplementation (mL/kg/d)	13.59 ± 4.91	8.98 ± 3.71	<0.001
Age to total enteral nutrition (d)	12.00 (5.00–71.00)	17.00 (8.00–42.00)	0.59
Parenteral nutrition time (d)	15.00 (3.00–94.00)	20.00 (7.00–48.00)	0.12
Caffeine	191 (75.49%)	40 (88.89%)	0.05
Respiratory support			0.005
No use	21 (8.33%)	4 (8.89%)	
Oxygen	9 (3.57%)	5 (11.11%)	
HFNC	3 (1.19%)	4 (8.89%)	
NCPAP	57 (22.62%)	11 (24.44%)	
BIPAP	102 (40.48%)	17 (37.78%)	
NIPPV	2 (0.79%)	0 (0.00%)	
IMV	58 (23.02%)	4 (8.89%)	
Duration of antibiotics			0.36
≤7 d	57 (22.62%)	5 (11.11%)	
≤14 d	69 (27.38%)	14 (31.11%)	
>14 d	126 (50.00%)	26 (57.78%)	
PS	162 (64.03%)	15 (33.33%)	<0.001
Delay to excrete meconium	65 (25.90%)	14 (31.11%)	0.47
Apnea	129 (50.99%)	23 (51.11%)	0.99
Septicemia before FI	18 (7.20%)	3 (6.67%)	0.90
Dolichasigmoid	8 (3.16%)	4 (8.89%)	0.07
PDA	14 (5.65%)	9 (20.45%)	<0.001
Thyroid dysfunction	143(91.67%)	118(86.77%)	0.18
Anemia	146 (57.71%)	30 (66.67%)	0.26
FI	136 (53.75%)	24 (53.33%)	0.96

HFNC, high flow nasal cannula; HMF, human milk fortifier; IMV, invasive mechanical ventilation; NCPAP, nasal continuous positive airway pressure; NIPPV, nasal intermittent positive pressure ventilation; PDA, patent ductus arteriosus; PS, pulmonary surfactant. *p* values from *t*-tests for continuous variables with normal distribution, nonparametric statistic tests for continuous variables with non-normal distribution and *X*^2^ tests for categorical variables.

**Table 3:** Univariate and multivariate logistic regression to assess the association of the HMF use with FI. Dates, 2015–2018.

HMF Use	*n* (%)	Unadjusted	Adjusted
		OR 95%CI *p* Value	OR 95%CI *p* Value
nonuse	45 (15.1%)	1.0	1.0
use	253 (84.9%)	1.02 (0.54, 1.92) 0.96	1.37 (0.24, 7.99) 0.72

FI, feeding intolerance; HMF, human milk fortifier. Adjusted for the caffeine; respiratory support; duration of antibiotics; pulmonary surfactant (PS); blood transfusion; septicemia before FI; dolichasigmoid; anemia; parenteral nutrition time; sex; placental abruption; gestational age (GA); birth weight (BW); 5 min Apgar score; placenta previa; admission temperature; age to start oral feeding; rate of milk supplementation; initial concentration of HMF; number of blood transfusion; patent ductus arteriosus (PDA); glucocorticoid; age of total enteral nutrition, rate of HMF supplementation.

**Table 4:** Univariate and multivariate logistic regression to assess the association of HM volume when HMF supplement with FI. Dates, 2015–2018.

HM Volume When HMF Supplement	*n* (%)	Unadjusted	Adjusted
		OR 95%CI *p* Value	OR 95%CI *p* Value
≥100 mL/kg/d	239 (94.47%)	1.0	1.0
<100 mL/kg/d	14 (5.53%)	5.57 (1.22, 25.39) 0.03	23.04 (1.67, 317.33) 0.02

FI, feeding intolerance; HM, human milk; HMF, human milk fortifier. Adjusted for caffeine; respiratory support; duration of antibiotics; pulmonary surfactant (PS); blood transfusion; delay to excrete meconium; apnea; septicemia before FI; anemia; parenteral nutrition time; placental abruption; gestational age (GA); birth weight (BW); 5 min Apgar score; placenta previa; gestational diabetes mellitus; admission temperature; age to start oral feeding; rate of milk supplementation; rate of HMF supplementation; initial concentration of HMF; glucocorticoid; age of total enteral nutrition.

**Table 5:** Univariate and multivariate logistic regression to assess the association of initial concentration of HMF with FI. Dates, 2015–2018.

Initial Concentration of HMF	*n* (%)	Unadjusted	Adjusted
		OR 95%CI *p* Value	OR 95%CI *p* Value
≤20 mg/mL	265 (89.2%)	1.0	1.0
>20 mg/mL	32 (10.8%)	1.49 (0.70, 3.17) 0.30	2.94 (1.02, 8.51) 0.04

FI, feeding intolerance; HMF, human milk fortifier. Adjusted for caffeine; respiratory support; septicemia before FI; parenteral nutrition time; gestational age (GA); 1 min Apgar score; admission temperature; rate of milk supplementation; rate of HMF supplementation; number of blood transfusion; patent ductus arteriosus (PDA); age of total enteral nutrition.

**Table 6:** Univariate and multivariate logistic regression to assess the association of rate of HMF supplementation with FI. Dates, 2015–2018.

Rate of HMF Supplementation	*n* (%)	Unadjusted	Adjusted
		OR 95%CI *p* Value	OR 95%CI *p* Value
−2~2 mg/mL/d	97 (33.45%)	1.0	1.0
2~5 mg/mL/d	96 (33.10%)	1.13 (0.64, 1.98) 0.68	3.77 (1.05, 13.57) 0.04
5~27 mg/mL/d	97 (33.45%)	0.90 (0.51, 1.58) 0.71	6.48 (1.79, 23.48) 0.004

FI, feeding intolerance; HMF, human milk fortifier. Adjusted for caffeine; respiratory support; duration of antibiotics; pulmonary surfactant (PS); blood transfusion; number of blood transfusion; apnea; septicemia before FI; dolichasigmoid; patent ductus arteriosus (PDA); anemia; parenteral nutrition time; gestational age (GA); birth weight (BW); 1 min Apgar score; placenta previa; admission temperature; age to start oral feeding; age of total enteral nutrition, rate of milk supplementation; HMF use; human milk (HM) volume when HMF supplement, initial concentration of HMF.

**Table 7:** Threshold effect analysis of the relationship between initial concentration of HMF and the risk of FI. Dates, 2015–2018.

Initial Concentration of HMF (mg/mL)	The Risk of FI	
	OR (95%CI)	*p* Value
Fitting model by standard linear regression	1.00 (0.94, 1.06)	1.00
Fitting model by two-piecewise linear regression		
Inflection point (K)	24	
<24 slope 1	0.93 (0.86, 1.01)	0.10
>24 slope 2	1.68 (1.01, 2.78)	0.04
Logarithm likelihood ratio test		0.01

HMF, human milk fortifier; FI, feeding intolerance. Adjusted for birth weight (BW); rate of milk supplementation; human milk (HM) volume when HMF supplement.

**Table 8:** Threshold effect analysis of the relationship between rate of HMF supplementation and the risk of FI. Dates, 2015–2018.

Rate of HMF Supplementation (mg/mL/d)	The Risk of FI	
	OR (95%CI)	*p* Value
Fitting model by standard linear regression	1.00 (0.95, 1.06)	0.92
Fitting model by two-piecewise linear regression		
Inflection point (K)	12.5	
<12.5 slope 1	0.93 (0.86, 1.01)	0.07
>12.5 slope 2	1.74 (1.08, 2.81)	0.02
Logarithm likelihood ratio test		0.002

HMF, human milk fortifier; FI, feeding intolerance. Adjusted for gestational age (GA); birth weight (BW); admission temperature; initial concentration of HMF.

## Data Availability

Data described in the article, code book, and analytic code will not be made available because the datasets analyzed in the present study are not publicly available, because the data are still being analyzed for other studies.
